# Effective Optical Properties of Inhomogeneously Distributed Nanoobjects in Strong Field Gradients of Nanoplasmonic Sensors

**DOI:** 10.1007/s11468-018-0769-4

**Published:** 2018-05-28

**Authors:** Krzysztof M. Czajkowski, Dominika Świtlik, Christoph Langhammer, Tomasz J. Antosiewicz

**Affiliations:** 1 Faculty of Physics, University of Warsaw, Pasteura 5, 02-093 Warsaw, Poland; 2 Centre of New Technologies, University of Warsaw, Banacha 2c, 02-097 Warsaw, Poland; 3 Department of Physics, Chalmers University of Technology, 412 96 Göteborg, Sweden

**Keywords:** Effective media, Maxwell Garnett approximation, Plasmonics, Sensors, Sintering, Nanoparticle layers

## Abstract

Accurate and efficient modeling of discontinuous, randomly distributed entities is a computationally challenging task, especially in the presence of large and inhomogeneous electric near-fields of plasmons. Simultaneously, the anisotropy of sensed entities and their overlap with inhomogeneous fields means that typical effective medium approaches may fail at describing their optical properties. Here, we extend the Maxwell Garnett mixing formula to overcome this limitation by introducing a gradient within the effective medium description of inhomogeneous nanoparticle layers. The effective medium layer is divided into slices with a varying volume fraction of the inclusions and, consequently, a spatially varying effective permittivity. This preserves the interplay between an anisotropic particle distribution and an inhomogeneous electric field and enables more accurate predictions than with a single effective layer. We demonstrate the usefulness of the gradient effective medium in FDTD modeling of indirect plasmonic sensing of nanoparticle sintering. First of all, it yields accurate results significantly faster than with explicitly modeled nanoparticles. Moreover, by employing the gradient effective medium approach, we prove that the detected signal is proportional to not only the nanoparticle size but also its size dispersion and potentially shape. This implies that the simple volume fraction parameter is insufficient to properly homogenize these types of nanoparticle layers and that in order to quantify optically the state of the layer more than one independent measurement should be carried out. These findings extend beyond nanoparticle sintering and could be useful in analysis of average signals in both plasmonic and dielectric systems to unveil dynamic changes in exosomes or polymer brushes, phase changes of nanoparticles, or quantifying light absorption in plasmon assisted catalysis.

## Introduction

Plasmonic sensing is a versatile tool, which uses strongly enhanced electric fields near the surface of metals to interrogate minute refractive index changes[1]. Its development has been made possible by breakthroughs in nanofabrication and chemistry, as well as by significant progress in our ability to calculate the optical properties of metallic nanostructures. These can, in principle, be divided into either single structures with various shapes, including dimers, trimers, or even more complex geometries [2, 3], and arrays of such elements in periodic, amorphous, or random arrangements. The complexity of modeling their optical properties, which is in focus here, varies with the structure and, in general, both single structures and their periodic lattices are relatively simple to model. However, once disorder is present [4]—and it is a common feature of many bottom up fabrication techniques [5] as well as in sensors in which stochastic processes take place [6, 7]—accurate and efficient modeling becomes challenging. A good example of such a modeling problem is an indirect nanoplasmonic sensing (INPS) experiment [8] in which a metal nanoantenna, whose immediate vicinity is filled with smaller nanoparticles [9], interrogates their material and/or chemical changes of and reports them via a peak shift [10, 11]. The challenge lies in the fact that the nanoparticles, which constitute the sensed element, are random in both size, position, and shape [12] and in simulations; it is all but impossible to model even a small fraction of plasmonic sensors interrogated in an actual experiment.

Simultaneously, while exact quantification of the optical properties of stochastically assembled materials is challenging, they have received significant attention in recent years. Pulse-laser nanostructuring has been used to convert Au films via heating and reshaping into stochastic surface enahnced Ramans scattering (SERS) substrates [13]. Random structuring is quite efficient at inducing scattering and coupling between nanoparticles and consequently may generate a broad spectral response, which can enhance the efficiency of solar harvesting via light trapping [14] or via antireflection coatings [15]. In addition to size, randomness may also be expressed via the shape of nanoparticles and enable ultra-broadband enhancement of nonlinear optical processes [16]. These examples highlight the need for accurate modeling of random nanostructures in strong electric fields.

A simple approach to study the optical properties of such complex systems [17] is to randomly generate an adequate number of potential realizations and average the computed quantity. While lacking finesse, this is, in fact, what is done in experiments with averaging implied when probing many elements simultaneously, and is indeed useful when microscopic statistics are sought [18]. In simulations, such approaches are, unfortunately, extremely resource-consuming and alternative means could potentially be useful. Moreover, when fine details are unimportant and the global response is sufficient, averaging over many simulation runs is wasteful and initial averaging is mandatory.

Such initial averaging of the optical response of randomly distributed dielectric inclusions in a host medium is known as an effective medium approach, in which the averaged permittivity describes the bulk response of the composite [19]. This solution is based on a more basic question of what is the effective/average polarizability of the medium [20]. In a quasistatic case of small particles compared to the wavelength, the properties of the effective medium are governed by the volume fraction of the inclusions. Naturally, more complex properties like anisotropy, chirality, and nonlinearities can also be included in effective descriptions [19]. Metal nanoparticles can similarly be homogenized/averaged into layers with effective properties [21–23]; however, due to comparatively larger polarizabilities than dielectric objects of similar (subwavelength) size, interaction between them may need to be taken into account [24]. Alternatively, when plasmonic particles become comparable to the wavelength of incident light, effective permittivity may no longer be well defined at a microscopic level. However, average properties of nanoparticles in layers [25, 26] or bulk [27] media can still be computed [28] or measured [29, 30].

Regardless of the physical complexity of a homogenized medium, one property should remain constant—the particles need to be isotropically (in a stochastic manner) arranged in the medium and, unless explicit retarded calculations are involved, the incident field should be uniform. These uniformities are, however, not a given. Specifically, in cases when the field exhibits strong gradients and nanoparticles, within it exhibit a nonuniform arrangement, homogenization into a uniform layer described by an effective permittivity, and may change the coupling conditions significantly. That is, potentially, the case in plasmonic sensing devices, since the surface wave supported by a metal interface exhibits fast spatial decay. For propagating plasmons, this decaying field may extend over hundreds of nanometers in the optical regime [31], while for the localized ones or tightly bound modes, the decay may only extend a few tens of nanometers [32, 33]. The localized surface plasmon resonance (LSPR) is only sensitive to events occurring or material present in the immediate vicinity of the metallic antenna, while changes even 20 nm away may hardly be registered. Under these conditions, homogenization of nanoparticles in the vicinity of the sensor into a single layer will tend to decrease the interaction efficiency between the plasmon and the object in its field, leading to an error in the estimation of signal changes in simulations. A question may be asked if homogenization is in fact necessary in the specific cases mentioned above. For very limited parameter studies, it may not. In general, however, use of an effective medium is beneficial, as it enables rapid analysis of a large parameter space without tedious averaging of optical cross-sections. Hence, to accurately model structures, such as noble metal nanodisks randomly decorated with nanoparticles [8, 10, 34], we propose the gradient effective medium (GEM), in which the effective permittivity incorporates a gradient with a spatial dependence derived from the inhomogeneous distribution of nanoparticles.

**Fig. 1:**
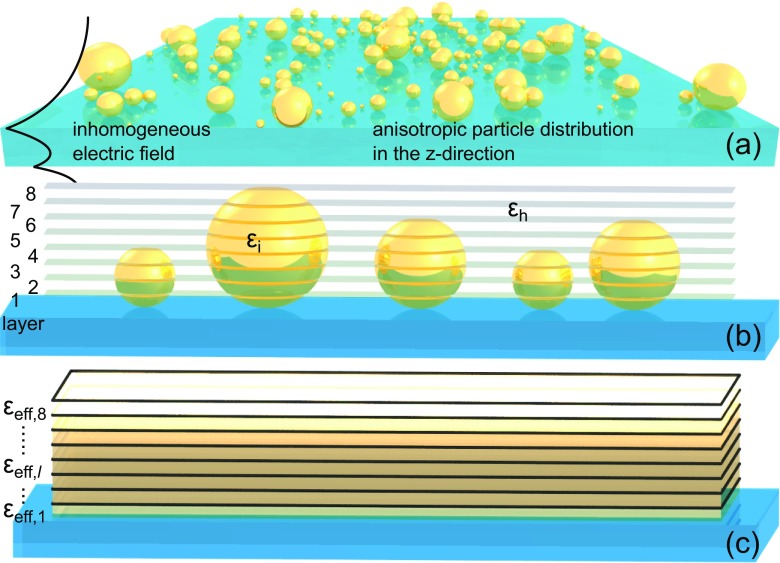
By converting the real mass distribution of particles into its distribution in layers, we can extend the effective medium theory to incorporate effects of a strongly varying electromagnetic field. **a** A layer of nanoparticles is deposited on a substrate resulting in an inhomogeneous distribution of mass in the direction normal to the substrate. When such a layer is in an electromagnetic field with a large gradient a single effective permittivity does not adequately describe the optical properties of the layer, as each particle interacts with a different field. **b** To preserve the mass distribution in a strongly varying field the nanoparticle layer is discretized into a number of sublayers beginning at the substrate and ending at the top of the largest nanoparticle. In each sublayer, the volume fraction occupied by the nanoparticles varies and is determined by their distribution. Here, we use the mean and standard deviation. **c** Each sublayer is homogenized with a unique volume fraction yielding a graded permittivity function in the direction normal to the substrate and what ensures that the probing field feels a mass distribution equivalent to that of a layer with discrete nanoparticles

A typical example where such an approach is useful, is nanoplasmonic sensors, which interrogate spatial changes of permittivity with a strongly inhomogenous field [35]. The active sensor elements, i.e., optical nanoantennas are on the order of up to a few hundred nanometers in size and made of a noble metal. Depending on the application area and operation environment of the sensor, it may also be coated by a dielectric spacer layer, which then supports smaller nanoobjects, such as catalyst nanoparticles, which are probed by the nanoantenna [8, 36]. In such, an INPS sensor, the nanoparticles are supported by a surface (as illustrated in Fig. 1), causes a non-negligible variation in mass (optical density) in the direction normal to the support layer. Consequently, the classical Maxwell Garnett (MG) mixing formula is not valid, especially since the nanopaticles are located in a strongly inhomogeneous plasmonic near-field. Such conditions occur for example in detection of exosomes [34], indirect nanoplasmonic sensing of hydrogen sorption by palladium [8], as well as sintering [37] of metal catalyst nanoparticles monitored in situ with LSPR [38] or plasmon assisted catalysis [2, 39–41]. With such a wide variety of sensing applications in need of accurate modeling, we expect that the gradient effective medium will be beneficial for rapid and accurate computational analysis.

This work is structured as follows. We begin by describing the gradient model—an extension of the classical MG effective medium approximation to account for spatial inhomogeneities in the inclusion (nanoparticle) distribution. Then, we perform initial validation of the model by studying the optical properties of inhomogeneous nanoparticle distributions supported by dielectric and metallic substrates. After this validation in simple planar systems, we employ the model to study LSPR measurements of sintering of Pd nanoparticles. We show the equivalence of averaging finite-difference time-domain (FDTD) simulation results for discrete nanoparticles and the gradient model, both of which reproduce the measured experimental evolution [10]. Finally, we apply the model to study the influence of microscopic variations in the size distributions of sintered nanoparticles and prove that the peak shift measured during this process is equally sensitive to not only the mean particle size, as demonstrated previously [10], but also to the particle dispersion and what complicates an unequivocal determination of state of the nanoparticles from a single measured value, i.e., the peak shift.

## Methods—Gradient Effective Permittivity Model

The properties of any material depend on its constituents and their spatial arrangement. In the case of a random structure, it is impractical to explicitly account for all interactions within its volume due to the complexity of the resulting equations as well as, and in practice more importantly, a lack of transference of the properties to other realizations of such a material with different microscopic internals. Hence, what is needed is a description of macroscopic properties which, at this higher level, skip over the exact internal nanostructuring and focus on global characteristics, such as material properties and fractional amounts of constituent materials, stochastic description of their distributions, etc. These few parameters, which characterize the heterogeneous material on a macroscopic level, are then used derive a homogenized, simpler macroscopic description of the properties of the ensemble [19].

### Maxwell Garnett Background

A macroscopic description of the effective electromagnetic properties of a heterogeneous medium involves creation of a permittivity function, which correctly accounts for how individual elements of the conglomerate interact with the external field. This implies that what is averaged is not the permittivities, but the fields and this requires careful accounting of the polarizabilities (*α*) of inclusions in the host medium. Assuming no interactions between inclusions, the effective permittivity *𝜖*_eff_ is given as [20]

1 $$ \frac{\epsilon_{\text{eff}}-\epsilon_{h}}{\epsilon_{\text{eff}}+ 2\epsilon_{h}}=\frac{4 \pi}{3}\sum\limits_{i} n_{i} \alpha_{i},  $$

where *α*_*i*_ is the polarizability of inclusion *i* and *n*_*i*_ is the particle density of species *i*. If we assume only one type of spherical inclusions, we then have in the quasistatic approximation that the polarizability *α* is expressed by the permittivity of host *𝜖*_*h*_ and inclusion *𝜖*_*i*_ media and its radius *r* (Fig. 1). For simplicity, one typically introduces the corresponding volume fraction of the inclusions as *δ* ≡ 4*πnr*^3^/3 [20],

2 $$ \frac{4 \pi}{3} n \alpha=\frac{\epsilon_{i}-\epsilon_{h}}{\epsilon_{i}+ 2\epsilon_{h}}\frac{4 \pi}{3} \mathit{n r}^{3}=\frac{\epsilon_{i}-\epsilon_{h}}{\epsilon_{i}+ 2\epsilon_{h}} \delta. $$

Combining the above with Eq. 1, yields the closed form of the Maxwell Garnett mixing formula

3 $$ \epsilon_{\text{eff}}^{\text{MG}}=\epsilon_{h}+ 3\delta\epsilon_{h}\frac{\epsilon_{i}-\epsilon_{h}}{\epsilon_{i}+ 2\epsilon_{h}-f(\epsilon_{i}-\epsilon_{h})}. $$

The MG formula utilizes only three parameters—the two permittivities and the volume fraction—to describe the macroscopic properties. While neither multiple scattering nor retardation are taken into account, these dependencies may be incorporated if needed. Hence, several extensions to the MG mixing formula have been proposed to account for various discrepancies introduced by using the effective medium approach [24, 42]. In these formulations, it is typically assumed that inclusions are distributed isotropically (on average) throughout the host medium and that they are exposed to a uniform field. However, this is not always the case, and the simple MG approach may fail when these conditions are not met, the problem we address in our work.

### Gradient effective permittivity (GEM) model

Here, we consider situations outside the limits of applicability of the classical MG formula, that is when anisotropically distributed inclusions within a host medium are illuminated by an inhomogeneous electric field. A typical example, where such conditions occur are thin nanoparticle layers supported by interfaces [12, 15, 43], where the presence of the interface breaks symmetry and introduces electric field gradients (see Fig. 1a). These can be generated by, e.g., reflection and interference on a dielectric substrate or the excitation of a surface plasmon polariton if the supporting material is metal.

**Fig. 2:**
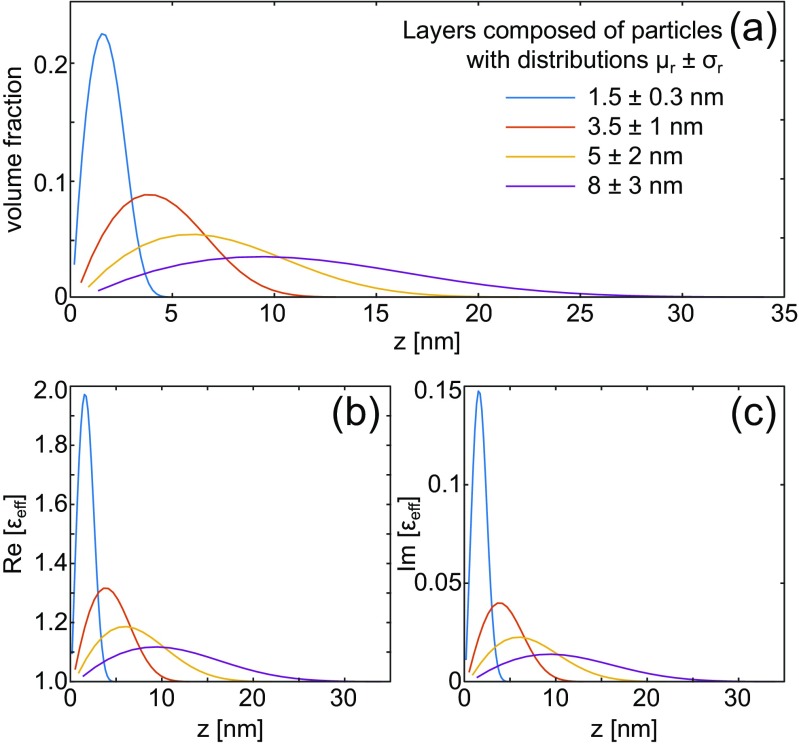
Exemplary results of homogenizing nanoparticle layers deposited on a substrate with its surface at *z* = 0 nm (cf. Fig. 1). The nanoparticles comprising a layer are defined by their mean radius and standard deviation (*μ*_*r*_ ± *σ*_*r*_). **a** Volume fraction of the nanoparticles (inclusions in effective medium wording) as function of the distance *z* from the interface and the **b** real and **c** imaginary parts of the graded effective permittivity for those layers. The volume fraction is maximized at the position of the mean nanosphere radius and, consequently, this determines the position of the maximum value of the permittivity. As the mean particle size increases, the center of mass moves away from the interface, resulting in a weaker interaction for bigger particles. The total graded medium thickness is equal to the diameter of the larges possible particle, which we assume is 2(*μ*_*r*_ + 3*σ*_*r*_)

In our proposed gradient effective medium approach (the GEM model), instead of homogenizing a nanoparticle layer into a single effective one like in an MG approximation, the nanoparticle layer is divided into several segments (thin sublayers) of equal thickness and each segment is homogenized separately (see Fig. 1b,c) using the classical MG mixing formula. The motivation behind such treatment is the fact that a supported nanoparticle layer consists, in principle, of objects of various size, Fig. 1a. By itself, this would not constitute a problem; however, the nanoparticles are not distributed equally in the direction normal (e.g., *z*-axis) to the substrate. Instead, they are placed at the interface. Hence, the average mass distribution is given by a particular function of *z* with parameters dependent on the particle distribution, c.f. an exemplary volume fraction dependence in Fig. 2a. When coupled with an inhomogeneous near-field of an LSPR, this function becomes important and needs to be taken into account in an effective treatment, which is what we do presently. We assume that the nanoparticles are sufficiently small to be described by the quasistatic polarizability, and we omit radiative coupling. For further simplification, no explicit interactions between layers are assumed within the model.

The key parameter in the GEM model, like in the MG approach, is the volume fraction *δ*—the ratio between the volume of inclusions, nanoparticles on the substrate, and the total volume of the layer. The distinction between the models is that the volume fraction is different in each sublayer and depends on the distance from the substrate as well as the distribution of particles in the layer. We base our initial analysis of nanoparticle layers on previously reported Pd particle distributions [10, 11], which are therein characterized by a mean and standard deviation. For convenience in calculations, we assume a Gaussian distribution of Pd nanospheres around a given mean radius *μ*_*r*_ with corresponding standard deviations *σ*_*r*_, although in reality a spherical shape may not be adequate. However, the general procedure is the same for any MG homogenized layer composed of individual nanoobjects, as in the GEM method the permittivity of each sublayer is based on the volume fraction of inclusions in that sublayer (and on the permittivities of the inclusions and the host medium).

The total thickness of the graded medium, which is equal to the largest assumed diameter given by *H* = 2(*μ*_*r*_ + 3*σ*_*r*_), is divided into *N*_*p*_ sublayers of thickness *h* = *H*/*N*_*p*_, see Fig. 1b. Each *l* th-sublayer contains a different amount of material *V*_*l*_ (of inclusions), which is given by a sum (integral) of all sphere segments (volume $V_{\text {seg},i}^{l}$) contained within this sublayer-*l* (Fig. 1b) over the radius distribution. The volume fraction of a sublayer *δ*_*l*_ can then be written as

4 $$ \delta_{l}=\frac{V_{l}}{V_{\text{sub}}}=\frac{{\sum}_{i} V_{\text{seg},i}^{l} {N_{i}^{l}}}{\mathit{s h}}=\frac{N_{p}}{H}\sum\limits_{i} V_{\text{seg},i}^{l} \frac{{N_{i}^{l}}}{s}, $$

where *i* runs over all sphere segments in a given sublayer of volume *V*_sub_ and *N*_*i*_ is the number of particles per layer area *s*. This allows us to write the expression for the effective permittivity of the *l* th-sublayer, see Fig. 1c,

5 $$ \epsilon_{\text{eff},l}=\epsilon_{h} \frac{2 \frac{N_{p}}{H} {\sum}_{i}{V^{l}_{\text{seg},i}\frac{{N^{l}_{i}}}{s}(\epsilon_{i}-\epsilon_{h})+\epsilon_{i}+ 2\epsilon_{h}}}{\epsilon_{i}+ 2\epsilon_{m}+\frac{N_{p}}{H} {\sum}_{i}{V^{l}_{\text{seg},i}\frac{{N^{l}_{i}}}{s}(\epsilon_{i}-\epsilon_{h})}}, $$

which is simply the MG formula applied independently to each slice of the homogenized graded medium. At this point and, indeed, throughout this work, we only consider a single material comprising the nanoparticles; however, the above equation can easily be expanded to include additional materials with different permittivities as is commonly reported in literature for the MG approximation [19]. This can be directly done by expanding the sums in Eq. 4 over index *i* to consider materials with different permittivities.

The volume fraction distribution, Fig. 2a, in each layer of nanoparticles, manifests itself in the effective dielectric function of the effective medium layer (see Fig. 2b, c). Both parts of the effective dielectric function increase with an increasing volume fraction, as plotted in Fig. 2. The sublayer with the largest volume fraction is the one which contains the middle segment of those spheres whose radii are close to the mean size *μ*_*r*_. The permittivity decreases with increasing distance from that sublayer, although in an asymmetric manner because sublayers close to the substrate contain contributions from both small and large particles, while sublayers beyond *μ*_*r*_ contain contributions only from larger ones.

A large particle size dispersion leads to a more uniform effective permittivity distribution across the gradient layer (e.g., 8 ± 3 nm), whereas nanoparticle layers with narrow radius distributions (1.5 ± 0.3 nm) are homogenized into gradient media with pronounced permittivity variation—the effect of different size distributions on *𝜖*_eff_ is shown in Fig. 2b, c. As the response of any electromagnetic system depends on the overlap of material and field, the manner in which the homogenization procedure is carried out affects the end result. As electromagnetic fields are rarely homogeneous, we expect that the gradient effective medium model will improve the accuracy of simulations over the MG approach, especially when considering strongly inhomogeneous electric fields. This improvement will be especially apparent for various types of surface plasmon sensors, which offer greatly enhanced, but quickly decaying, fields near the metal-dielectric interface.

### Numerical Calculations

In order to test the gradient model, we use interchangeably the transfer matrix method (TMM) and the FDTD method. The former are used in initial evaluation of discontinuous nanoparticle layers supported by flat substrates, in cases in which the nanoparticles are homogenized and the structure is planar. The latter are used in every case in which discrete nanoparticles are simulated as well as when computing the optical response of an LSPR sensor decorated with discrete or homogenized nanoparticles.

Modeled structures are placed on a substrate with a refractive index of 1.45. The parameters of discontinuous Pd nanoparticle layers are initially based on those fabricated for the work of Adibi et al. [10], but subsequently tested mean radii and their standard deviations are probed in a broad range, as can be observed in other experiments [12]. The initial equivalent mass thickness is assumed to be 0.5 nm [10]. In calculations, the permittivity of Pd, as well as of Ag when needed for the LSPR sensor, is taken from Palik [44], while for Au from Johnson and Christy [45]. FDTD calculations are carried out using FDTD Solutions from Lumerical, Inc., Canada. Due to the presence of nanoparticles with sizes down to 2 nm in diameter, a mesh resolution of 0.5 nm is used. As the plasmon resonance of such small Pd nanoparticles is far outside the considered wavelength range, the accuracy, which is enhanced by the use of subpixel averaging, is adequate.

In the case of nanoparticles supported by flat substrates, we use periodic boundary conditions in the transverse directions, while in simulations of nanoplasmonic sensing perfectly matched layer absorbing boundary conditions are used. The simulation area is 200 × 200 nm^2^. In simulations with discrete nanoparticles, the support surface is uniformly decorated with non-overlapping nanoparticles with randomly drawn positions. For FDTD, we generate a set of positions with radii being drawn from an appropriate Gaussian distribution with mean radius *μ*_*r*_ and its standard deviation *σ*_*r*_. In TMM, we generate a vector of discrete radius values ranging from zero to three standard deviations. Then, the number of particles per area is calculated for each radius value based on the Gaussian distribution normalized so that the total particle volume corresponds to an equivalent mass thickness of 0.5 nm.

## Results and Discussion

### Nanoparticle Arrays on Flat Dieletric Substrates

We begin by investigating the behavior of a Pd nanoparticle layer on a flat dielectric substrate with the TMM to assess the influence of the size distribution of nanoparticles in layer on its optical properties and quantify the accuracy of the gradient effective medium approximation in comparison to rigorous calculations with FDTD. As the electric fields under such conditions do not have very strong field gradients, we expect moderate differences between using the proposed GEM averaging method and a classical MG approach. However, recent investigations of thermal dewetting of thin Au films into a nanoparticle layer [12] or the feasibility of antireflection coatings based on silver nanoparticle films [15] motivate our choice and showcase the realm of applicability of the GEM method.

**Fig. 3:**
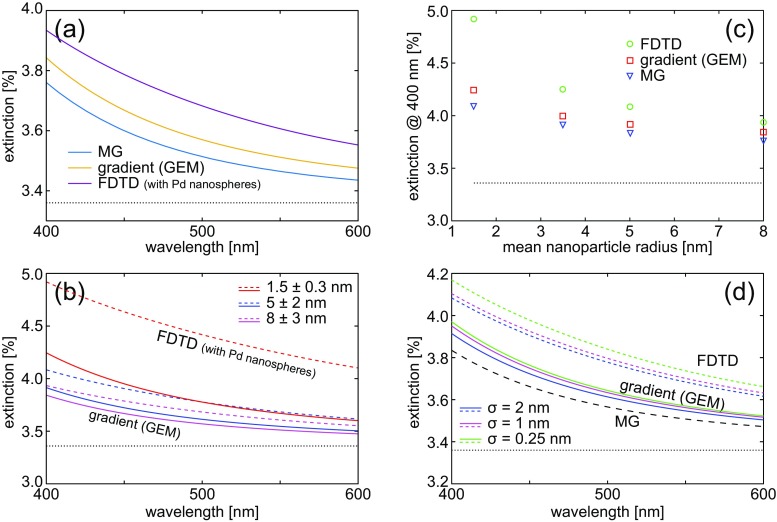
Comparison of optical properties of layers of nanoparticles calculated using three methods: the introduced gradient effective medium (GEM), classical MG, and FDTD with explicit nanoparticles. **a** Extinction of a Pd nanoparticles with *r* = 8 ± 3 nm on a substrate with *n* = 1.45 obtained with (blue) the MG approximation, (orange) the gradient model, and (purple) FDTD with discrete nanoparticles. **b** Extinction of nanoparticle layers with selected radius distributions. Solid lines: GEM, dashed: FDTD. **c** Extinction of sintered Pd nanoparticles with increasing mean size. **d** Extinction spectra for Pd nanoparticles with varying standard deviation of particle radius with *μ*_*r*_ = 5 nm. Solid lines—GEM, dashed— FDTD, black dashed line—MG. In all plots, the black dotted line marks extinction of a bare *n* = 1.45 substrate. The GMG approximation gives results closer to the FDTD-derived ones than the MG approach. Layers with large and sparse particles are better described by the gradient homogenized model as a result of a smaller impact of interparticle coupling

The extinction spectrum of an examplary nanoparticle layer (*r* = 8 ± 3 nm) on a dielectric substrate is presented in Fig. 3a. As the total amount of metal is small, extinction is mainly determined by the substrate (dotted line in Fig. 3 at 3.36%); however, the contribution from the Pd nanoparticles is visible in all approaches. The full FDTD calculation with discrete nanospheres shows an extinction increase of up to 0.5%, Fig. 3a. The effective medium approaches underestimate it, although the GEM model offers a more accurate description than the MG one.

Next, in Fig. 3b, we look into how the optical properties evolve with a change of the radius distribution of the Pd nanoparticles. The initial distribution, which corresponds to an as-deposited Pd layer with an equivalent thickness of 0.5 nm and contains nanoparticles with a size distribution of *r* = 1.5 ± 0.3 nm, undergoes a hypothetical sintering process based on Adibi et al. [10], thereby increasing its mean radius and standard deviation. This causes the homogenized layer’s thickness to grow and effectively decreases the volume fraction, making the Pd layer increasingly more transparent, as evident in Fig. 3b from the FDTD simulations with explicit nanoparticles. This result shows that while the amount of matter is the same, the size distribution cannot be neglected, and that, as a whole, a dense layer of small nanoparticles interacts with light most efficiently [46]. This evolution is also captured by the gradient model, although the degree of change is smaller than in FDTD with explicit nanospheres. Extinction is underestimated by both effective medium approaches (GEM and MG), because in the present, formulation radiative coupling between the particles is neglected. This effect is most prominent with dense layers of small particles. During sintering, the mean nanoparticle size increases during coalescence and the number density decreases. This causes a decrease of interparticle scattering and leads to increased accuracy, as evident in Fig. 3c. We expect that including multiple scattering in the effective permittivity will increase the accuracy.

The improved validity of the GEM model for simulating the optical response of nanoparticle layers stems from accounting for particle size distribution by accurately capturing the spatial dependence of the volume fraction. This is because the effective permittivity profile in the gradient model depends on both the mean and standard deviation of the nanoparticle radius. The influence of the latter (*σ*_*r*_) is presented in Fig. 3d. Gradient layers become effectively less dense with increasing size dispersion and leads to decreased extinction by lowering the maximal refractive index value. A similar effect is also observed when the *μ*_*r*_ increases when the total nanoparticle mass is kept constant (Fig 3c).

### Nanoparticle Arrays on Flat Metal Substrates (SPR)

**Fig. 4:**
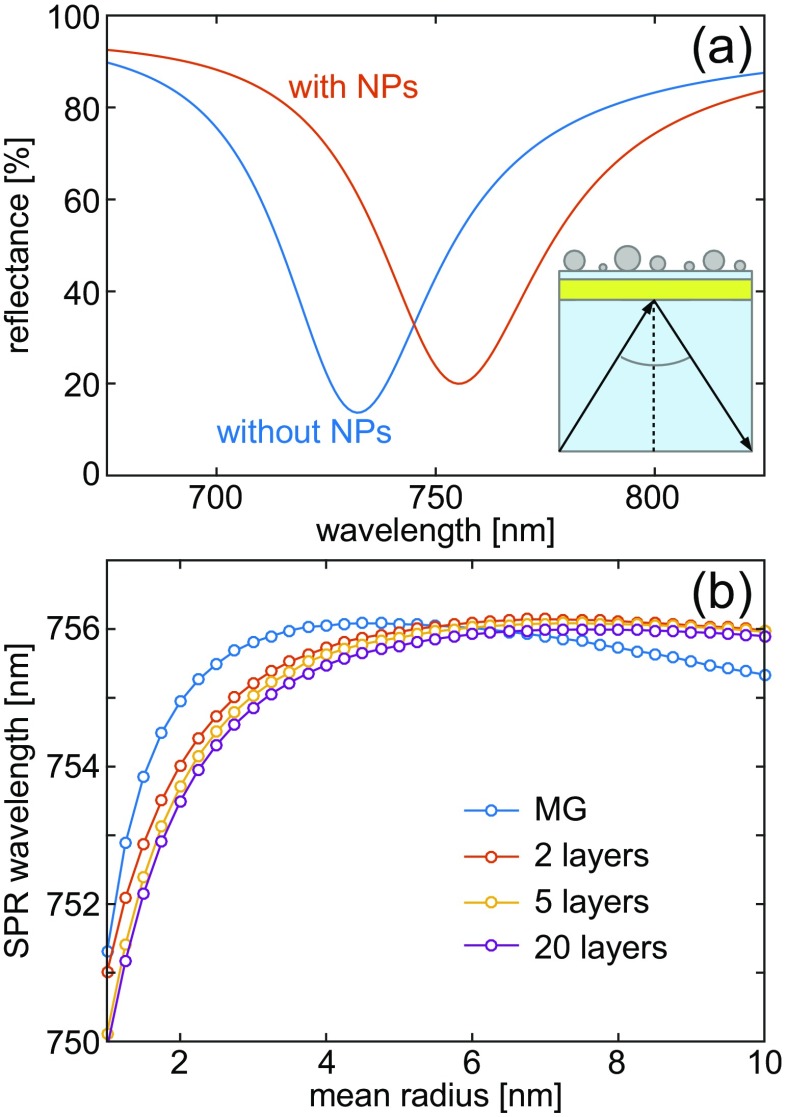
Modeling of surface plasmon polaritons in the presence of a nanoparticle layer. **a** The SPP shifts to the red due to the presence of nanoparticles, but **b** due to a large field enhancement and gradient of the SPP the simulation is sensitive to the homogenization method. The classical MG model is off by more than 1 nm for certain nanoparticle distributions and already a two-layer GEM yields significant improvement. The GEM model converges quickly with an increasing number of layers

Pure dielectric planar structures do not offer strong electric fields nor large gradients. These appear at the surface plasmon resonance (SPR), which we study for a 60-nm Au layer deposited on a glass substrate with a constant refractive index of 1.45 (the Au is capped by a 10 nm 1.45 dielectric; see inset in Fig. 4a). In the Kretschmann configuration at 46^∘^ angle of incidence, the resonance position is at 732.2 nm without any Pd, while upon depositing a nanoparticle layer (*μ*_*R*_ = 1.5 nm), the reflection dip shifts by ca. 20 nm, see Fig. 4a. As FDTD calculations with discrete nanoparticles are not feasible for the required accuracy, we only compare the MG and gradient models. While in both cases, in general, a red-shift of the peak position is observed with an increase of *μ*_*R*_, a discrepancy between the MG and gradient model is clearly visible in Fig. 4b. The MG effective medium approach predicts a maximum peak shift of ca. 5 nm for *μ*_*R*_ ≈ 4.5 nm, followed by a blue-shift of 1 nm when the mean particle size increases further. When we calculate the same evolution with the gradient model, the SPR-vs-*μ*_*R*_ dependence is different. Already with two sublayers, the maximum peak shift occurs at a larger *μ*_*R*_, and the reversal of the initial red-shift is much smaller. With increasing discretization, similar behavior is observed, although the influence of the number of sublayers converges. The main origin of this behavior is that a single MG layer effectively moves an amount of material away from the metal interface by averaging it out over an increasingly thicker layer. In the gradient model, the contribution from small nanoparticles to the SPR shift is preserved as their optical density is closer to the metal surface, where the plasmon intensity is the strongest and most sensitive to any variations.

### Nanoparticle Arrays Probed by a Nanoplasmonic Sensor (LSPR)

The general investigations reported above demonstrate that the gradient model is more accurate than the classical MG. Hence, we now use the gradient model to simulate localized plasmonic sensing of nanoparticle layers undergoing sintering [10]. The electric field and its gradient of the LSPR are larger than for the dielectric substrate or the SPR sensor described above, being also the reason for the high sensitivity of the INPS sensor. The additional justification of using the GEM approach, in this case, is the fact that one simulation with a gradient layer corresponds to an averaged response of many experimentally measured or simulated sensors with explicitly simulated nanoparticles. Hence, it significantly simplifies and quickens numerical analysis.

**Fig. 5:**
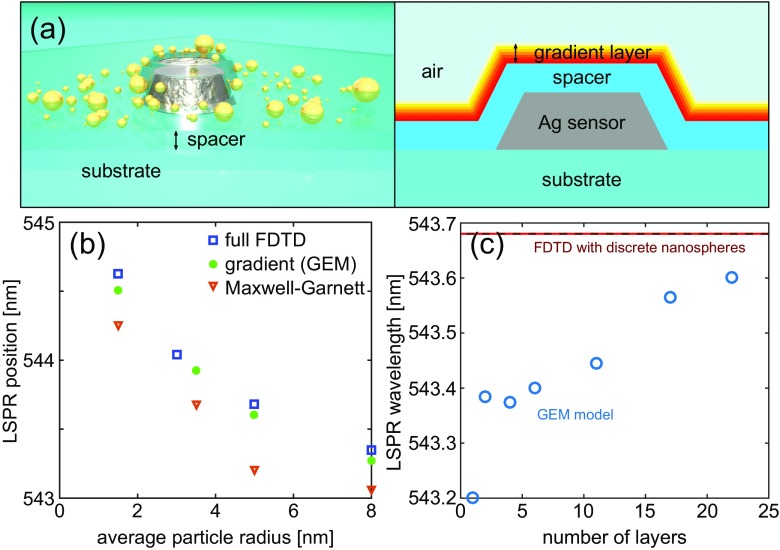
Indirect nanoplasmonic sensing of nanoparticle sintering. **a** FDTD simulation structure with discrete Pd nanospheres probed by an Ag sensor and a corresponding model with a GEM description of the nanoparticle layer used to calculate subsequent results. **b** LSPR peak positions of an Ag sensor probing a hypothetical sintering process (i.e., surface-energy driven coalescence and growth of small nanoparticles into larger ones) of Pd nanoparticles. The Pd nanoparticles are treated as either discrete nanospheres (squares), a GEM layer (circles), or a classical MG medium (triangles). The GEM yields much better accuracy than the MG model when compared to explicitly modeled nanospheres with FDTD. **c** Convergence of the LSPR peak position computed for a gradient effective medium with a variable number of layers for *r* = 5 ± 2 nm. To obtain sub-0.1 nm accuracy, 22 effective medium sublayeres 1-nm thick are required

Figure 5a shows how a discrete Pd nanoparticle distribution is transformed into a gradient layer. In a typical INPS sensor arrangement [8], the plasmonic antenna (here 25-nm thick, 30-nm radius) is isolated from the environment by a dielectric spacer (10 nm, *n* = 1.48), which supports the probed nanoobjects. Here, we discretize the gradient layer into 1-nm thick sublayers and, as after averaging they have (qualitatively), a permittivity reminiscent of a lossy dielectric, 0.5-nm meshing is adequate (cf. Fig. 2b, c).

In FDTD calculations, we compare the predicted LSPR peak evolution under hypothetical sintering based on experimental observations [10] employing the traditional MG approach of a single layer, our GEM model, and explicit Pd nanoparticles (averaged spectra over 100 realizations in each case). In every case, the calculations yield a blue-shift of the resonance wavelength for increasing particle radius as a consequence of decreased nanoparticle volume fraction, as shown in Fig. 5b (i.e., material moves away from the sensor). This behavior is consistent with experimental measurements [10]. However, the MG approach with a single effective permittivity, overestimates the progression of the peak shift by as much as 0.5 nm. While in absolute numbers, this is not a significant difference, the whole sintering process is characterized by peak shifts on the order of 1 nm; hence, the relative discrepancy of the MG approach is significant. In contrast, the gradient effective permittivity is within 0.1 nm of the peak shift evolution for explicitly defined nanospheres, offering very good accuracy and justifying its use in predictive studies with a computational (time) burden decreased by an order of magnitude.

**Fig. 6:**
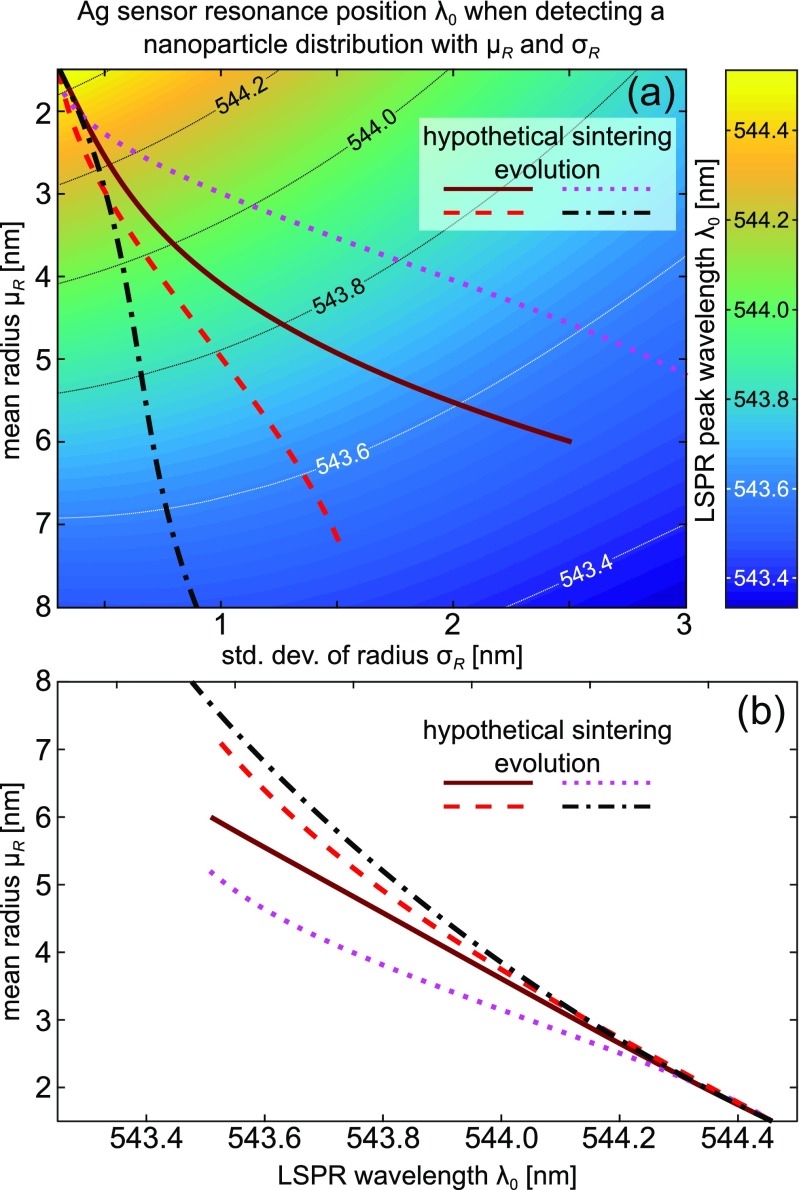
Application of GEM method to study plasmonic sensing of Pd nanoparticle sintering. **a** The colormap shows the peak position *λ*_0_ of an Ag sensor (see Fig. 5a) coated by a layer Pd nanoparticles of varying distributions (*μ*_*R*_, *σ*_*R*_) for a constant mass per unit area (0.5 nm equivalent thickness). Observe, that any one value of *λ*_0_ corresponds to a set of mean radii and their deviations. During sintering both *μ*_*R*_ and *σ*_*R*_ evolve and, as just shown using the GEM, a given resonance position does not uniquely identify the state of the nanoparticle layer. To highlight this, we designate hypothetical nanoparticle evolutions with different (*μ*_*r*_,*σ*_*r*_) dependencies, which begin from the same distribution but terminate at different states, which are characterized by approximately the same peak shift of ca. 0.9 nm. **b** We plot the simulated peak shifts on the *x*-axis and mark their corresponding mean radii on the *y*-axis. The measured peak shifts are the same in each case (544.4 → 543.5 nm), and one can see the ambiguity in elucidating the state of the nanoparticle layer without access to the standard deviation. Hence, an experiment measuring only one quantity cannot differentiate among the possible final mean particle distributions. Such an analysis was feasible only with the GEM approach and not with averaging many simulation results with explicitly defined nanoparticles

Before employing the new approach to investigate sintering in detail, we present a convergence test in Fig. 6a for a nanoparticle layer with *r* = 5 ± 2 nm. The LSPR peak position clearly depends on the number of homogenization layers. For a single layer (MG approach), we have a 0.6-nm mismatch, which decreases more than sixfold when using 22 effective layers each 1-nm thick. Any further improvement in accuracy can probably be obtained by including interaction within the MG mixing formulas, but for the present work sub-0.1 nm accuracy is adequate. Consequently, it is possible to study the correlation between nanoparticle size distribution and LSPR peak position using the mean and standard deviation as parameters describing the distribution and what was not feasible with rigorous FDTD calculations in a realistic time frame (a single vs. hundreds of simulations, each a few hours long).

We now move beyond the study of Adibi et al. [10] and extend the analysis of plasmonic sensing of nanoparticle sintering to a broad range of *μ*_*R*_ and *σ*_*R*_ parameters and calculate the resonance position of an Ag sensor when decorated by a given distribution. We assume in the calculation that the parameters *μ*_*r*_ and *σ*_*r*_ are independent of each other and *μ*_*r*_ ∈ [1.5,8] nm and *σ*_*r*_ ∈ [0.3,3] nm. The results, plotted in Fig. 6a using the colormap, clearly demonstrate, that the resonance wavelength is determined by both the average particle size and its dispersion. Hence, without knowledge of how the size dispersion is correlated with the mean radius during sintering, it may be impossible to accurately determine the state of the layer of nanoparticles from a single peak position readout. This highlights the importance of accurate calibration studies and a potential lack of transferal of peak shift vs. mean particle size for various devices/conditions.

To highlight the importance of the standard deviation in addition to the mean particle size, we devise four hypothetical sintering experiments, which follow different size/dispersion relations from a common initial state of *r* = 1.5 ± 0.3 nm and terminate at different states described by the same relative peak shift Δ*λ* = 0.9 nm vs. that of the initial state. These evolution pathways, marked in Fig. 6a with the four thick lines, exhibit different rates of increasing mean radius and its standard deviation, favoring rapid increase of the former (black), the letter (pink), or relatively balanced (remaining two). If these evolution pathways are plotted as Δ*λ* vs. *μ*_*r*_ and *σ*_*R*_, the relative differences are clear. However, in an experiment the readily available quantity is the peak shift and if only that quantity is used—as in Fig. 6b—then an ambiguity in the interpretation of the data is very clear. This result clearly underlines the fact, that a given resonance position does not uniquely identify the state of the nanoparticle layer, and additional data must be gathered. It can come from prior knowledge of the sintering process (*μ*_*R*_(*t*) and *σ*_*R*_(*t*), *t*—time) or from additional, simultaneous measurements. This conclusion is, of course, applicable to any sensing study of supported nanoparticle layers with a certain size distribution and will be in fact even more relevant if the shape of the nanoparticles is not uniform.

## Summary and Conclusions

Electromagnetic mixing formulas provide a simple route to describe the effective properties of inhomogenous materials. Here, we have introduced a gradient effective medium, GEM, approximation to provide a more accurate description of thin layers composed of supported nanoparticles in inhomogeneous electric fields than possible with a standard Maxwell Garnett approximation. The usefulness GEM stems from the fact that nanoparticles in supported layers are not placed isotropically throughout the volume in question, but have a defined distribution in the direction normal to the substrate interface. The model captures this distribution and combines it with the effects of strong electromagnetic field gradients present in such a layer, as is typical for (nano)plasmonic sensors. In this work, we place emphasis on quantifying the response of LSPR sensors to changes in nanoparticle layers with the ultimate goal of being capable of determining the evolution of the size distribution of nanoparticles in the layer during a sintering process [10, 11, 47]. However, the GEM approach is general enough that it can also be used efficiently for dielectric substrates, as recently employed for antireflection coatings based on silver nanoparticle films [15] or when using nanoparticle films for sensing [12].

One of the most important conclusions of our work is that the detected signal coming from nanoparticle layers depends strongly on *both* the mean and standard deviation of the particle distribution. This means that predictive numerical and/or analytical studies of corresponding experimental systems will yield ambiguous results. Hence, quantifying the state of a nanoparticle array (e.g., average size) may not be possible using a single readout like the peak shift. This complication indicates that in-depth studies of sensor systems or expanded analysis methods are probably necessary. These can be realized quickly and reliably with numerical methods incorporating the GEM to model the average properties of nanoparticle layers at significantly reduced computational cost compared to rigorous simulations with explicitly defined nanoparticles.

We also would like to mention that aside from the particle size and distribution influence on the measured signal, the nanoparticle shape should also be considered. In this work, we used only spherical nanoparticles throughout the entirety of the sintering evolution; however, this may not be the case. In fact, the slower saturation of the LSPR with mean size in modeling when compared with experiment [10] may likely be attributed to mostly horizontal growth rather than vertical[48–50]. While this aspect was not considered in our analysis of sintering, the gradient model is general enough to homogenize an layer composed of arbitrarily shaped nanoparticles. The GEM is also able to incorporate effects related to different permittivities of homogenized nanoparticles, i.e., simultaneous presence of two species of sensed nanoobjects. At a more general level, we have also demonstrated that the accuracy of the gradient layer approach increases with an increasing finesse of the graded effective medium, and we suggest that a possible route to further improve the accuracy of the graded model is the incorporation of the effects of direct scattering between nanoparticles or mediated by nearby interfaces for inclusions in thin layers [43].

Finally, it is worthwhile mentioning that the applicability of the gradient model extends beyond nanoparticle sintering studies with plasmonic sensors, considered here as a case to benchmark our modeling approach, or general evolution of the shape and size of nanoparticles comprising a thin film [12]. In principle, whenever an average signal over many sensors with strongly inhomogeneous fields is collected—and when the detected objects do not exhibit perfect homogeneity—the presented model is a likely candidate to be used. This can include detection of dynamic changes in exosomes [34], indirect nanoplasmonic sensing of hydrogen sorption by palladium [8], probing of polymer blushes [51], studying plasmoelectric effects in nanoparticles [30], or in modeling light absorption effects in plasmon-assisted catalysis [2, 39–41].
